# Statin Utilization Patterns and Outcomes for Patients with Acute Coronary Syndrome During and Following Inpatient Admissions

**DOI:** 10.1007/s10557-018-6800-3

**Published:** 2018-05-31

**Authors:** Susan H. Boklage, Elisabetta Malangone-Monaco, Lorena Lopez-Gonzalez, Yao Ding, Caroline Henriques, Joseph Elassal

**Affiliations:** 1 Regeneron Pharmaceuticals, Inc., 777 Old Saw Mill River Rd., Tarrytown, NY 10591 USA; 2 Truven Health Analytics, Bethesda, MD USA

**Keywords:** Acute coronary syndrome, Statin, Cardiovascular events

## Abstract

**Purpose:**

High-intensity statins (HIS) are recommended by current treatment guidelines for patients with clinical atherosclerotic cardiovascular disease and should be administered soon after an acute coronary syndrome (ACS) event and maintained thereafter. However, adherence to guidelines remains adequate. Statin utilization patterns during index hospitalization and the first year after ACS event, and the association between statin utilization and post-discharge clinical and economic outcomes, are described.

**Methods:**

Retrospective, observational study of US adults from the MarketScan Research Databases (2002–2014) with ≥ 1 inpatient admission for ACS and no evidence of previous ACS event < 12 months prior to index.

**Results:**

In total, 7802 patients met inclusion criteria. The most common index hospitalization primary diagnosis was myocardial infarction (94.6%). In the 3-month period before ACS admission, 3.4 and 14.9% of patients received HIS or low-to-moderate intensity statin, versus 13.2 and 30.7% during index hospitalization, and 16.4 and 45.1% in the year of follow-up. Of 1336 patients with a statin prescription filled on/after discharge, 53.2% filled prescriptions within 15 days of discharge and 14.9% delayed for > 91 days. The most common post-index hospital admissions for cardiovascular events were due to recurrent ACS (incidence rate = 115.2), heart failure (110.0), and revascularization (76.4). During follow-up, 2355 patients (30.2%) had all-cause inpatient admissions and 1136 (14.6%) had cardiovascular-specific admissions; mean all-cause medical and healthcare costs were $2456 and $2870, respectively, per patient per month.

**Conclusions:**

Statin dosing and utilization of HIS remains lower than recommended in current treatment guidelines, leaving patients at considerable risk of subsequent cardiovascular events.

**Electronic supplementary material:**

The online version of this article (10.1007/s10557-018-6800-3) contains supplementary material, which is available to authorized users.

## Introduction

Acute coronary syndrome (ACS) refers to a spectrum of clinical symptoms compatible with acute myocardial ischemia and includes the diagnosis of unstable angina and myocardial infarction (MI) with or without ST elevation [1, 2]. In 2010, the estimated number of unique hospitalizations for ACS in the USA was 1,141,000 [3]. The estimated annual cost of ACS was $150 billion in the USA in 2008 [2].

Following an initial ACS event, patients are at high risk of recurrent cardiovascular events [4–6]. Reducing low-density lipoprotein cholesterol (LDL-C) with statin therapy reduces the risk of cardiovascular events in high-risk patients with atherosclerotic cardiovascular disease (ASCVD) [7–10]. Prior placebo-controlled studies have shown beneficial effects of low-to-moderate intensity statins (LMIS) [11, 12]. In the PROVE-IT trial of patients with a recent ACS event, an intensive lipid-lowering statin regimen (high-intensity statins [HIS]) provided greater protection against death or major cardiovascular events than a standard LMIS regimen [7].

HIS therapy is recommended by the 2013 American College of Cardiology (ACC) and American Heart Association (AHA) guidelines, and the 2004 updated Adult Treatment Panel (ATP III) guidelines, for patients with clinical ASCVD (including ACS), and should be administered soon after an ACS event and maintained thereafter [13, 14].

Adherence to recommended treatment guidelines should translate to an improvement in the survival and quality of life of ACS patients; however, adherence remains inadequate [2]. There is a paucity of real-world data on guideline-directed statin use among ACS patients during hospitalization. The present study sought to describe statin utilization patterns among ACS patients during index hospitalization and in the first year after the ACS event and assess the association between statin utilization and post-discharge clinical and economic outcomes.

## Methods

### Data Source

This was a retrospective, observational US study of ACS patients using data from the MarketScan® Inpatient Drug Link File, comprising the MarketScan Commercial and Medicare Supplemental databases. ACS index hospitalization was defined as the first ACS hospitalization event occurring between January 1, 2002 and December 31, 2014.

The MarketScan Commercial Claims database and MarketScan Medicare Supplemental database both comprised inpatient and outpatient medical information as well as outpatient prescription drug claims, covered under various health plans between 1995 and 2014. The MarketScan Hospital Drug Database comprised hospital billing information from 695 acute care hospitals in the USA and included 65.6 million hospital discharges between January 2002 and December 2014. The MarketScan Inpatient Drug Link file matched patients from the MarketScan Commercial, Medicare Supplemental, and Medicaid databases to hospital discharge records in the MarketScan Hospital Drug Database, using a match key comprising hospital identifier, admission and discharge date, principal diagnosis, and patient age and sex. Non-unique key values were excluded*.* This method has been used in previous studies [15].

### Study Population and Design

Patients aged ≥ 18 years with at least one inpatient admission (index) for an ACS (defined as MI or unstable angina [International Classification of Diseases, Ninth Revision, Clinical Modification (ICD-9-CM) codes 410.xx or 411.1x] requiring hospitalization between January 1, 2002 and December 31, 2014) were included in the analysis. Continuous enrollment with medical and pharmacy benefits for ≥ 12 months pre-index ACS admission (baseline) was required. Patients with a previous ACS admission in the 12 months prior to the index date were excluded.

The study comprised a 12-month pre-index (baseline) period, the ACS inpatient stay, and a variable-length follow-up period (Supplementary Fig. 1). The follow-up period extended from the discharge date for the index admission until the earliest of 12 months post-discharge, inpatient death, dis-enrollment from health insurance, or end of study period (December 31, 2014).

### Data Analysis

Demographics, clinical and hospitalization characteristics at baseline, statin treatment patterns, and outcomes were summarized descriptively. It was therefore not necessary to describe statistical analysis methods.

#### Statin Use and Intensity, and Lipid-Lowering Therapy Treatment Pattern, During the ACS Hospitalization

A binary variable was created to indicate whether patients received any statins. Based on statin intensity, patients were divided into LMIS and HIS categories (Supplementary Table 1). Based on the proportion of days covered (PDC), defined as the number of days in the observation period covered by medication divided by the number of days in the observation period, patients were categorized into (i) HIS, (ii) LMIS, (iii) monotherapy non-statin lipid-lowering therapy (LLT) treatment, (vi) mixed treatment (for those with evidence of multiple intensities of statins, i.e., no single statin intensity with PDC ≥ 50%), or (v) no treatment (for those with a PDC ≥ 50% for no statin treatment). Duration of statin/non-statin LLT use (total number of days between the first and last service date for the therapy or the discharge date) was calculated.

#### Statin Use and Treatment Pattern During Follow-Up

Statin medications dispensed within 30 days of the ACS hospitalization discharge date were considered continued use of statin therapies. Patients met the criteria for statin use during follow-up if they had their pre-index statin supply available on the ACS discharge date and into the follow-up period, and/or ≥ 1 outpatient prescription claim for any statin during the follow-up period. Patients were assigned into a treatment category if the PDC was ≥ 50%.

Statin treatment patterns were categorized as persistent if patients remained in the same LLT category during follow-up without exceeding a gap of 15 days following the last supply date of the last observed claim for statin medication in each intensity category. Discontinuation (during follow-up) was defined as absence of prescription claim for 15 days or more following the last supply date for the statin intensity administered during the index admission.

#### Cardiovascular Outcomes, Healthcare Utilization, and Healthcare Costs

The incidence rates of recurrent ACS, stroke, heart failure (secondary to MI), revascularization, and cardiovascular-specific deaths (identified from the ICD-9-CM codes; Supplementary Table 2) were reported up to 12 months post-index. Incidence rates were calculated by dividing the number of patients experiencing at least one occurrence of an event during follow-up by the total number of days between the ACS discharge date and the event of interest, or censoring at the end of follow-up for patients without an event. For patients with multiple cardiovascular-specific inpatient admissions during the follow-up period, the first inpatient date and/or emergency room date was selected. Rates were multiplied by 1000 to present as 1000 person-years.

All-cause and cardiovascular-specific healthcare utilization were reported by type of service (inpatient, outpatient, and pharmacy). The follow-up period was capped at 12 months for patients with more than 12 months of follow-up. To account for the variable-length follow-up, the counts of admissions, days, visits, or services and prescriptions were presented as per patient per month (PPPM) units.

Healthcare costs were based on allowed amounts of adjudicated claims, including insurer and health plan payments, as well as patient cost-sharing in the form of copayments, deductibles, and coinsurance. All costs were adjusted for inflation using the healthcare consumer price index and standardized to the year 2014 US dollars.

## Results

### Patient Demographic and Clinical Characteristics at Baseline (Pre-Index ACS Hospitalization)

**Fig. 1:**
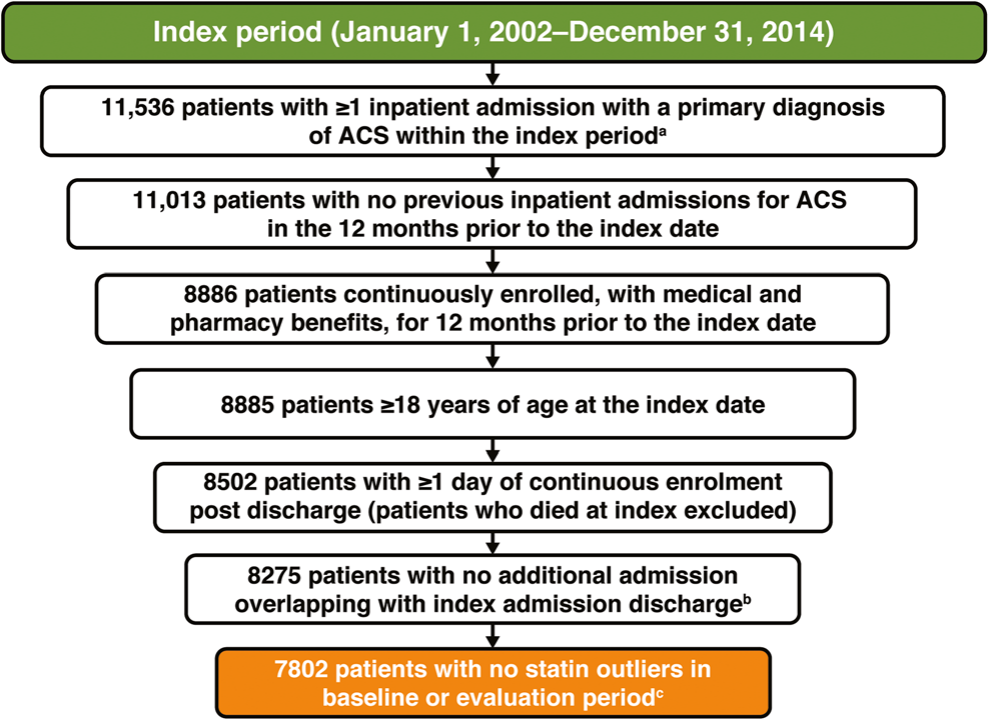
Patient flow. *ACS* acute coronary syndrome. Superscript a indicates that the first observed inpatient admission for ACS occurring during this period (between January 1, 2002 and December 31, 2014) was recorded as the index date, provided that there was no evidence of a previous ACS event in the 12 months prior to this time. Superscript b indicates a hospital transfer. Superscript c indicates that patients were excluded if statin doses administered during the inpatient stay were below the lowest available or above the highest available doses

Of the 11,536 patients who had at least one inpatient admission with primary diagnosis of ACS, 7802 (67.6%) met the inclusion criteria (Fig. 1). The mean age was 66.7 years, 2650 (34.0%) were women, and 4241 (54.4%) had Medicare (Table 1). The most common comorbid conditions prior to index hospitalization were hypertension (45.3%), diabetes (24.9%), and dyslipidemia (22.9%).

**Table 1:** Patients’ demographic and clinical characteristics at baseline (pre-index ACS hospitalization)

	Study cohort *N* = 7802
Age, mean years (SD)	66.7 (12.8)
Gender, *n* (%)	
Male	5152 (66.0)
Female	2650 (34.0)
Geographic US region, *n* (%)	
Northeast	131 (1.7)
North central	2251 (28.9)
South	5163 (66.2)
West	240 (3.1)
Unknown	17 (0.2)
Payer, *n* (%)	
Commercial	3561 (45.6)
Medicare	4241 (54.4)
Comorbid conditions, *n* (%)	
Hypertension	3531 (45.3)
Diabetes	1946 (24.9)
Dyslipidemia	1788 (22.9)
Valvular heart disease	740 (9.5)
Heart failure	704 (9.0)
Stroke	575 (7.4)
Unstable angina	505 (6.5)
Atrial fibrillation	493 (6.3)
Chronic kidney disease/renal impairment	490 (6.3)
Revascularization	327 (4.2)
Percutaneous coronary intervention	250 (3.2)
History of MI	136 (1.7)
Coronary artery bypass graft	40 (0.5)
Cardiovascular medications, *n* (%)	
Any statin^a^	2382 (30.5)
Beta-blockers	2190 (28.1)
Angiotensin-converting enzyme inhibitor	1613 (20.7)
Diuretics	1586 (20.3)
Calcium channel blockers	1555 (19.9)
Angiotensin receptor blockers	1152 (14.8)
Antiplatelet agents	791 (10.1)
Non-statin LLT	648 (8.3)
Anticoagulants	377 (4.8)

*ACS* acute coronary syndrome, *LLT* lipid-lowering therapy, *MI* myocardial infarction, *SD* standard deviation

^a^Patients could have had more than one statin in the 12-month pre-index hospitalization period

Before their index event, 2382 patients (30.5%) had received a filled claim for statin therapy with 1946 (81.7%) being dispensed LMIS. Based on fill patterns and corresponding PDC categorization, in the 3 months prior to index, 267 (3.4%), 1162 (14.9%), and 6141 (78.7%) patients were assigned to HIS, LMIS, and no statin treatment, respectively.

### Clinical Characteristics and LLT Treatment Patterns During ACS Index Hospitalization and Follow-Up Period

The most common index hospitalization primary diagnosis was MI (*n* = 7377; 94.6%), including non-ST-segment elevation MI (*n* = 4174; 53.5%), ST-segment elevation MI (*n* = 2882; 36.9%), and other MI (*n* = 313; 4.0% [Supplementary Fig. 2]).

A total of 3905 (50.1%) and 863 patients (11.1%) underwent percutaneous coronary intervention and coronary artery bypass during index hospitalization, respectively. The use of cardiovascular medications during index hospitalization was higher overall than at baseline, with the most common being anticoagulants (93.4%), beta blockers (89.4%), antiplatelet agents (76.7%), and statins (70.9%; Supplementary Fig. 2), compared with 4.8, 28.1, 10.1, and 30.5%, respectively, at baseline.

**Fig. 2:**
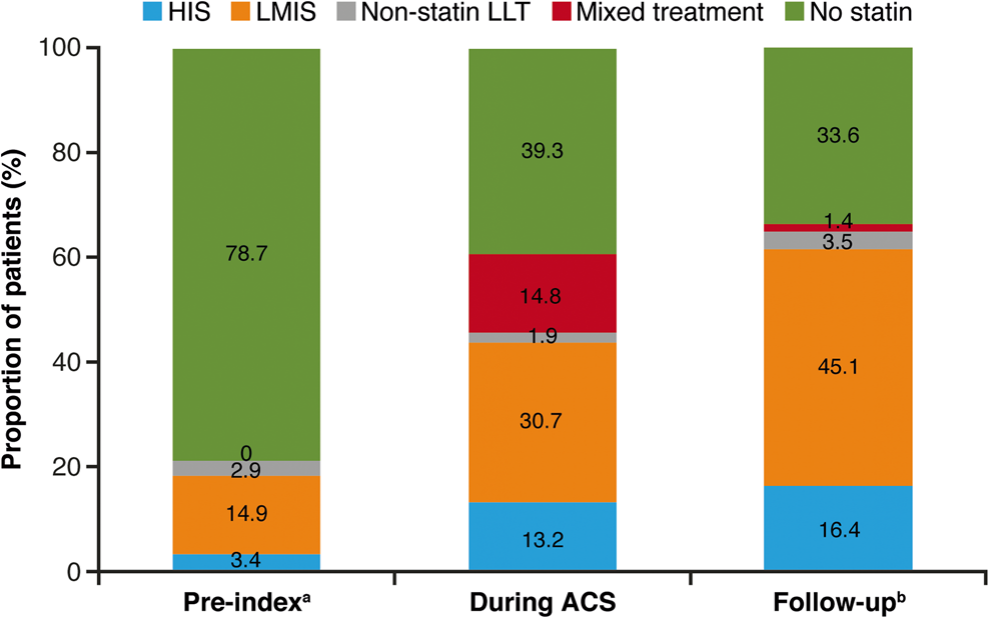
Statin treatment patterns in all patients by PDC ≥ 50% (*N* = 7802). *ACS* acute coronary syndrome, *HIS* high-intensity statin, *LLT* lipid-lowering therapy, *LMIS* low-to-moderate-intensity statin, *PDC* proportion of days covered. Superscript a identifies therapies assigned according to PDC in the 3-month pre-index hospitalization. Superscript b indicates that statins were prescribed in the outpatient setting for 64% of all patients

In the 3-month period immediately before ACS admission (based on PDC ≥ 50% categorizations), 267 and 1162 patients (3.4 and 14.9%) received HIS or LMIS, respectively; however, these increased to 1028 (13.2%) and 2396 (30.7%) during index ACS hospitalization, and 1282 (16.4%) and 3519 (45.1%) in the year of follow-up (Fig. 2). In the year of follow-up, 6060 patients (77.7%) filled ≥ 1 statin prescription. Of the 5528 patients using any statin at index hospitalization, 4958 patients (89.7%) filled a prescription for any statin treatment in the outpatient setting during the year of follow-up or had pre-index carryover statin supply available at the time of discharge.

A total of 4468/4958 patients (90.1%) filled a prescription for any statin within 30 days of index hospitalization discharge or had pre-index carryover statin supply available at the time of discharge. Of these, during the follow-up period, a similar proportion experienced changes in statin treatment regimen (3.8% from LMIS to HIS and 3.9% from HIS to LMIS), whereas 2137 patients (47.8%) had no modifications.

A sub-analysis categorizing statin treatment patterns into the periods 2002–2005 (*n* = 2917) and 2006–2014 (*n* = 4885), to examine the potential impact of the PROVE-IT study [7], showed that HIS was received by 1017 patients (20.8%) with an ACS event during 2006–2014 compared with 265 (9.1%) during 2002–2005 (Supplementary Fig. 3).

**Fig. 3:**
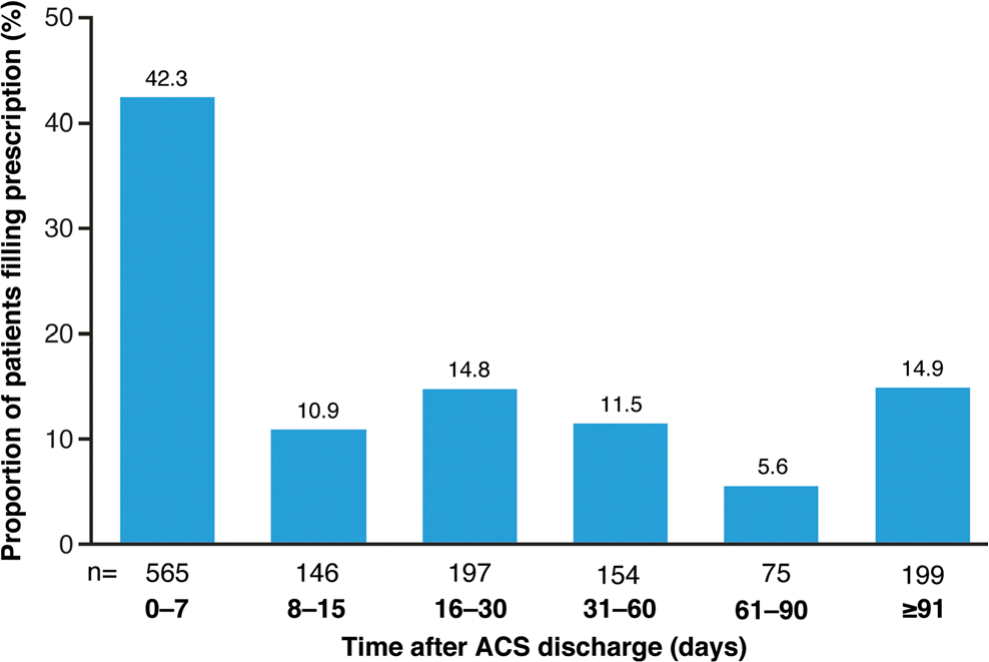
Proportion of patients filling statin prescriptions by time after ACS discharge (*N* = 1336^a^). *ACS* acute coronary syndrome, *SD* standard deviation. Superscript a indicates the number of patients with any statin prescription filled on or after ACS discharge, excludes patients with pre-index carryover statin at the time of discharge. Mean (SD) time to first statin prescription fill was 42.1 (71.3) days

Of the 1336 patients with any statin prescription filled on or after ACS discharge (i.e., excluding patients with pre-index hospitalization carryover statin supply available at the time of discharge), 711 (53.2%) filled prescriptions within 15 days of being discharged and 199 (14.9%) delayed for > 91 days (Fig. 3). Overall, 1182 patients (26.5%) discontinued statin treatment in the follow-up year.

### Clinical and Economic Outcomes Following an ACS Inpatient Event (Follow-Up Period)

Following an ACS inpatient event, the most common post-index hospital admissions for cardiovascular events were due to recurrent ACS (incidence rate 115.2/1000 person-years), heart failure (110.0), and revascularization (76.4). Incidence rates of MI and unstable angina-related admissions were 72.5 and 61.7, respectively; the incidence rate for cardiovascular-specific inpatient deaths was 11.4 (Supplementary Table 3).

During the follow-up period, 2355 patients (30.2%) had an all-cause inpatient admission and 1136 (14.6%) had cardiovascular-specific inpatient admissions (Table 2). The mean all-cause PPPM medical and healthcare (medical + outpatient pharmacy) costs were $2456 and $2870, respectively; the corresponding values for cardiovascular-specific costs were $1111 and $1391, respectively (Supplementary Table 4). Inpatient services, and outpatient and pharmacy costs, were the key drivers of healthcare costs.

**Table 2:** All-cause and cardiovascular-specific healthcare utilization outcomes during the follow-up period

	All discharges, *N* = 7802	
	All-cause	Cardiovascular-specific^a^
Any inpatient admissions, *n* (%)	2355 (30.2)	1136 (14.6)
Duration to first inpatient admissions or censoring, mean days (SD)	262 (130.3)	290 (119.4)
Outpatient services, *n* (%)		
Emergency room	3266 (41.9)	1297 (16.6)
Physician office	7131 (91.4)	6411 (82.2)
Laboratory	5740 (73.6)	2619 (33.6)
Radiology	5934 (76.1)	2969 (38.1)
Other	7505 (96.2)	6420 (82.3)
Outpatient pharmacy	7393 (94.8)	7231 (92.7)

*ICD-9-CM* International Classification of Diseases, Ninth Revision, Clinical Modification, *SD* standard deviation

^a^Cardiovascular-specific healthcare utilization and costs were defined by pulling medical claims with ICD-9-CM diagnosis codes for a cardiovascular condition and pharmacy claims for cardiovascular medications. For inpatient admissions, the diagnosis code must be in the primary diagnosis position on the claim

## Discussion

Despite evidence that treatment with statins, especially HIS, reduces the risk of cardiovascular events in high-risk patients with ASCVD [8], results from the present study showed that a considerable proportion of patients did not receive any statin during index hospitalization. HIS was underutilized during, and in the year following, the index ACS event. HIS was received by a greater proportion of patients with an index ACS event in 2006–2014 (21%) than 2002–2005 (9%), potentially due to the impact of results from the PROVE-IT study [5, 7]; however, utilization remained lower than is recommended in current treatment guidelines [13, 16, 17]. Of note, the 2013 ACC/AHA guideline [13] did not apply at the time that many of the patients in this study were being treated. These patients would have received statin treatment as recommended in the 2002 ATP III guidelines, which were developed when there was less evidence of the benefit of HIS therapy [18], before publication of the PROVE-IT study [7]. There was, however, a growing body of evidence demonstrating a benefit of early initiation of statin therapy in patients with ACS. The MIRACL study, highlighted in the ATP III guidelines, demonstrated that statin treatment initiated in the hospital in patients with non-Q MI or unstable angina was safe and associated with a 16% relative risk reduction at 16 weeks [19]. The ATP III guidelines update published in 2004 [14] acknowledged the PROVE-IT study; nevertheless, 39.3 and 33.6% of patients from the present study did not receive statin during the index hospitalization or follow-up, respectively.

Studies have shown low adherence to, and suboptimal dosing of, statins in patients at high risk of cardiovascular events in the USA [20–22]. For example, in a study of 273,308 ASCVD patients, only 8.8% had received HIS, 29.5% had received LMIS, and 61.7% were non-statin users [21]. Similarly, of 23,040 recent ACS patients with a filled statin prescription, only 27% received HIS [23]. The proportion of patients prescribed HIS in a study of 117,989 patients hospitalized for an MI was higher than that observed in our study (in 2014, the first filled prescription after discharge was HIS in 71.7% of those aged 19–64 years and 57.5% of those aged 66–75 years [24]). However, adherence to treatment post-discharge in a similar cohort of MI patients was low, with only 41.6% remaining highly adherent to HIS after 2 years [22]. Suboptimal statin therapy is a frequent factor limiting LDL-C goal attainment among high-risk patients in the USA. Suggested reasons for suboptimal statin dosing include statin intolerance, lack of acceptance of guideline recommendations, and contraindications [20, 21, 23].

Our findings, along with published data [20, 21, 23], underscore a major issue with both underutilization and non-adherence to HIS, and the potential health consequences caused. Data have shown that, in patients with recent ACS, HIS provided greater protection against death or major cardiovascular events than LMIS [7], emphasizing the need for adherence to guideline-directed statin use in ACS [13]. In a recent study, older age, female sex, renal dysfunction, and heart failure during hospital admission were the most common baseline characteristics of non-use of “high-potency statins” [25]. Factors that have been linked with an increased likelihood of being prescribed a HIS in high CV risk patients include male sex [21], no previous statin use [26, 27], younger age [28], presence of hypertension [28], and receiving prescription from a cardiologist [22]. However, the main drivers of statin underutilization and non-adherence post-ACS events need further examination. The present study showed that recommendations for a rapid and maintained treatment of high-risk patients with HIS were generally not followed, with 47% of patients delaying filling their prescription for > 15 days following discharge from an ACS event, despite accommodations for those on statins before the event.

In this study, following an ACS inpatient event, the highest hospital admission incidence rates were recurrent ACS, heart failure, and revascularization. The cardiovascular event rates observed during the follow-up period were in keeping with a previous report showing high 1-year cardiovascular risk of 15.1% in recent ACS patients [6]. The overall cardiovascular-specific healthcare costs in the present study contributed to 48% of the overall PPPM total healthcare expenditure. Underutilization of statins in this study lends further support to previously published data that adherence to statins is inadequate [2]. Better use of statins as per recommendations may improve clinical outcomes and reduce economic burdens from patients with ACS. Considering the high incidence of recurrent events observed on a background of statin therapy in this study, add-on treatment with other lipid-lowering therapies such as ezetimibe or proprotein convertase subtilisin/kexin 9 (PCSK9) inhibitors may also be necessary in this patient population, particularly in those failing to reach LDL-C treatment targets with statins, in accordance with recent guidelines [29, 30]. Recent results from a large outcomes study have demonstrated significant reductions in major adverse cardiovascular events with the PCSK9 inhibitor alirocumab in a population of patients who had experienced an ACS event 1–12 months prior to initiation of treatment [31].

### Limitations

While the evidence to support representation of the administrative claims data has been well established, as this is linked with inpatient drug utilization, there may be inherent biases with this linkage that negatively impact the ability to generalize these results to all regions and practices. However, the use of linked data could be considered a strength as it allows individuals to be followed from inpatient to outpatient settings. These findings are also strengthened by the consistency of this sample with other published studies, with a similar proportion of patients with non-ST-segment elevation MI compared with ST-segment elevation MI to that reported in the literature [32–34].

Results from the present study do not provide information on the rationale for prescribing statin therapy at different doses, or for not prescribing statin therapy. While pharmacy claims show the amount of medication a patient was dispensed, it is not known whether patients filling their prescriptions take medications as directed. The data from this study are subject to data coding limitations and data entry error.

## Conclusions

Utilization of HIS remains lower than is recommended in current treatment guidelines [13], with poor rates of adherence in patients with recent ACS, consequently leaving patients at considerable risk of subsequent cardiovascular events. The data from this study show that there is opportunity to improve statin utilization during and following an ACS event. Further investigation to determine drivers of statin therapy underutilization during index ACS event and thereafter is warranted.

## Electronic Supplementary Material

ESM 1 (DOCX 257 kb)

## References

[1] Kumar A, Cannon CP (2009). Acute coronary syndromes: diagnosis and management, part I. Mayo Clin Proc.

[2] Kolansky DM (2009). Acute coronary syndromes: morbidity, mortality, and pharmacoeconomic burden. Am J Manag Care.

[3] Mozaffarian D, Benjamin EJ, Go AS, Arnett DK, Blaha MJ, Cushman M (2016). Heart disease and stroke Statistics-2016 update: a report from the American Heart Association. Circulation.

[4] Motivala AA, Tamhane U, Ramanath VS, Saab F, Montgomery DG, Fang J, Kline-Rogers E, May N, Ng G, Froehlich J, Gurm H, Eagle KA (2008). A prior myocardial infarction: how does it affect management and outcomes in recurrent acute coronary syndromes?. Clin Cardiol.

[5] Murphy SA, Cannon CP, Wiviott SD, McCabe CH, Braunwald E. Reduction in recurrent cardiovascular events with intensive lipid-lowering statin therapy compared with moderate lipid-lowering statin therapy after acute coronary syndromes from the PROVE IT-TIMI 22 (Pravastatin or Atorvastatin Evaluation and Infection Therapy-Thrombolysis In Myocardial Infarction 22) trial. J Am Coll Cardiol 2009;54(25):2358–2362. 10.1016/j.jacc.2009.10.005 20082923

[6] Navar AM, Steen DL, Wojdyla D, Sanchez RJ, Khan I, Peterson E, Pencina M (2017). Recurrent cardiovascular event rates in a contemporary cohort of 829,498 adults with atherosclerotic cardiovascular disease. J Am Coll Cardiol.

[7] Cannon CP, Braunwald E, McCabe CH, Rader DJ, Rouleau JL, Belder R, Joyal SV, Hill KA, Pfeffer MA, Skene AM, Pravastatin or Atorvastatin Evaluation and Infection Therapy-Thrombolysis in Myocardial Infarction 22 Investigators (2004). Intensive versus moderate lipid lowering with statins after acute coronary syndromes. N Engl J Med.

[8] Cholesterol Treatment Trialists’ (CTT) Collaborators, Mihaylova B, Emberson J, Blackwell L, Keech A, Simes J, et al. The effects of lowering LDL cholesterol with statin therapy in people at low risk of vascular disease: meta-analysis of individual data from 27 randomised trials. Lancet. 2012;380(9841):581–90. 10.1016/S0140-6736(12)60367-5 PMC3437972 22607822

[9] Cannon CP, Blazing MA, Giugliano RP, McCagg A, White JA, Theroux P, Darius H, Lewis BS, Ophuis TO, Jukema JW, de Ferrari GM, Ruzyllo W, de Lucca P, Im K, Bohula EA, Reist C, Wiviott SD, Tershakovec AM, Musliner TA, Braunwald E, Califf RM, IMPROVE-IT Investigators (2015). Ezetimibe added to statin therapy after acute coronary syndromes. N Engl J Med.

[10] Blazing MA, De Lemos JA, Dyke CK, Califf RM, Bilheimer D, Braunwald E (2001). The A-to-Z trial: methods and rationale for a single trial investigating combined use of low-molecular-weight heparin with the glycoprotein IIb/IIIa inhibitor tirofiban and defining the efficacy of early aggressive simvastatin therapy. Am Heart J.

[11] Heart Protection Study Collaborative Group (2002). MRC/BHF heart protection study of cholesterol lowering with simvastatin in 20,536 high-risk individuals: a randomised placebo-controlled trial. Lancet.

[12] Shepherd J, Blauw GJ, Murphy MB, Bollen EL, Buckley BM, Cobbe SM (2002). Pravastatin in elderly individuals at risk of vascular disease (PROSPER): a randomised controlled trial. Lancet.

[13] Stone NJ, Robinson JG, Lichtenstein AH, Bairey Merz CN, Blum CB, Eckel RH, Goldberg AC, Gordon D, Levy D, Lloyd-Jones DM, McBride P, Schwartz JS, Shero ST, Smith SC, Watson K, Wilson PWF (2014). 2013 ACC/AHA guideline on the treatment of blood cholesterol to reduce atherosclerotic cardiovascular risk in adults: a report of the American College of Cardiology/American Heart Association Task Force on Practice Guidelines. J Am Coll Cardiol.

[14] Grundy SM, Cleeman JI, Merz CN, Brewer HB, Jr., Clark LT, Hunninghake DB et al. Implications of recent clinical trials for the National Cholesterol Education Program Adult Treatment Panel III guidelines. Circulation 2004;110(2):227–239. 10.1161/01.CIR.0000133317.49796.0E 15249516

[15] Montejano L, Vo L, McMorrow D (2016). Transitions of care for people with type 2 diabetes: utilization of antihyperglycemic agents pre- and post-hospitalization. Diabetes Ther.

[16] Jacobson TA, Ito MK, Maki KC, Orringer CE, Bays HE, Jones PH, McKenney JM, Grundy SM, Gill EA, Wild RA, Wilson DP, Brown WV (2015). National lipid association recommendations for patient-centered management of dyslipidemia: part 1—full report. J Clin Lipidol.

[17] Catapano AL, Graham I, De Backer G, Wiklund O, Chapman MJ, Drexel H (2016). 2016 ESC/EAS guidelines for the management of dyslipidaemias. Eur Heart J.

[18] National Cholesterol Education Program Expert Panel on Detection (2002). Evaluation and treatment of high blood cholesterol in adults. Third report of the National Cholesterol Education Program (NCEP) Expert Panel on detection, evaluation, and treatment of high blood cholesterol in adults (adult treatment panel III) final report. Circulation.

[19] Schwartz GG, Olsson AG, Ezekowitz MD, Ganz P, Oliver MF, Waters D, Zeiher A, Chaitman BR, Leslie S, Stern T, Myocardial Ischemia Reduction with Aggressive Cholesterol Lowering (MIRACL) Study Investigators (2001). Effects of atorvastatin on early recurrent ischemic events in acute coronary syndromes: the MIRACL study: a randomized controlled trial. JAMA.

[20] Lin I, Sung J, Sanchez RJ, Mallya UG, Friedman M, Panaccio M, Koren A, Neumann P, Menzin J (2016). Patterns of statin use in a real-world population of patients at high cardiovascular risk. J Manag Care Spec Pharm.

[21] Huang Q, Grabner M, Sanchez RJ, Willey VJ, Cziraky MJ, Palli SR, Power TP (2016). Clinical characteristics and unmet need among patients with atherosclerotic cardiovascular disease stratified by statin use. Am Health Drug Benefits.

[22] Colantonio LD, Huang L, Monda KL, Bittner V, Serban MC, Taylor B, Brown TM, Glasser SP, Muntner P, Rosenson RS (2017). Adherence to high-intensity statins following a myocardial infarction hospitalization among Medicare beneficiaries. JAMA Cardiol.

[23] Steen DL, Khan I, Becker L, Foody JM, Gorcyca K, Sanchez RJ (2017). Patterns and predictors of lipid-lowering therapy in patients with atherosclerotic cardiovascular disease and/or diabetes mellitus in 2014: insights from a large US managed-care population. Clin Cardiol.

[24] Rosenson RS, Farkouh ME, Mefford M, Bittner V, Brown TM, Taylor B, Monda KL, Zhao H, Dai Y, Muntner P (2017). Trends in use of high-intensity statin therapy after myocardial infarction, 2011 to 2014. J Am Coll Cardiol.

[25] Eisen A, Cannon CP, Braunwald E, Steen DL, Zhou J, Goodrich EL, Im KA, Dalby AJ, Spinar J, Daga S, Lukas MA, O’Donoghue ML (2017). Predictors of nonuse of a high-potency statin after an acute coronary syndrome: insights from the stabilization of plaques using Darapladib-thrombolysis in myocardial infarction 52 (SOLID-TIMI 52) trial. J Am Heart Assoc.

[26] Rosenson RS, Kent ST, Brown TM, Farkouh ME, Levitan EB, Yun H, Sharma P, Safford MM, Kilgore M, Muntner P, Bittner V (2015). Underutilization of high-intensity statin therapy after hospitalization for coronary heart disease. J Am Coll Cardiol.

[27] Valentino M, Al Danaf J, Panakos A, Ragupathi L, Duffy D, Whellan D (2016). Impact of the 2013 American College of Cardiology/American Heart Association cholesterol guidelines on the prescription of high-intensity statins in patients hospitalized for acute coronary syndrome or stroke. Am Heart J.

[28] Virani SS, Woodard LD, Akeroyd JM, Ramsey DJ, Ballantyne CM, Petersen LA (2014). Is high-intensity statin therapy associated with lower statin adherence compared with low- to moderate-intensity statin therapy? Implications of the 2013 American College of Cardiology/American Heart Association cholesterol management guidelines. Clin Cardiol.

[29] Lloyd-Jones DM, Morris PB, Ballantyne CM, Birtcher KK, Daly DD Jr, et al. 2016 ACC expert consensus decision pathway on the role of non-statin therapies for LDL-cholesterol lowering in the Management of Atherosclerotic Cardiovascular Disease Risk A Report of the American College of Cardiology Task Force on Clinical Expert Consensus Documents. J Am Coll Cardiol. 2016;68(1):92–125. 10.1016/j.jacc.2016.03.519 27046161

[30] Landmesser U, Chapman MJ, Stock JK, Amarenco P, Belch JJF, Boren J (2017). 2017 update of ESC/EAS task force on practical clinical guidance for proprotein convertase subtilisin/kexin type 9 inhibition in patients with atherosclerotic cardiovascular disease or in familial hypercholesterolaemia. Eur Heart J.

[31] Steg P. Cardiovascular outcomes with alirocumab after acute coronary syndrome: results of the ODYSSEY outcomes trial. Presented at the 67th Annual Scientific Session of the Am Coll Cardiol (ACC), 10–12 March 2018 (Presentation number 401–08). Orlando; 2018.

[32] Khera S, Kolte D, Aronow WS, Palaniswamy C, Subramanian KS, Hashim T, Mujib M, Jain D, Paudel R, Ahmed A, Frishman WH, Bhatt DL, Panza JA, Fonarow GC (2014). Non-ST-elevation myocardial infarction in the United States: contemporary trends in incidence, utilization of the early invasive strategy, and in-hospital outcomes. J Am Heart Assoc.

[33] Rogers WJ, Frederick PD, Stoehr E, Canto JG, Ornato JP, Gibson CM, Pollack CV, Gore JM, Chandra-Strobos N, Peterson ED, French WJ (2008). Trends in presenting characteristics and hospital mortality among patients with ST elevation and non-ST elevation myocardial infarction in the National Registry of myocardial infarction from 1990 to 2006. Am Heart J.

[34] Shah B, Bangalore S, Gianos E, Liang L, Peacock WF, Fonarow GC, Laskey WK, Hernandez AF, Bhatt DL (2014). Temporal trends in clinical characteristics of patients without known cardiovascular disease with a first episode of myocardial infarction. Am Heart J.

