# Supplementary material for: Statin Utilization Patterns and Outcomes for Patients with Acute Coronary Syndrome During and Following Inpatient Admissions
Source: Cardiovasc Drugs Ther. 2018 May 31;32(3):273–80. doi: 10.1007/s10557-018-6800-3 (PMC6018577; doi:10.1007/s10557-018-6800-3)
Supplement: Supplementary file 1 — (DOCX 257 kb) [file 10557_2018_6800_MOESM1_ESM.docx]

# Supplementary Material

Supplementary Table 1. Classification of statin therapy

| **Statin** | **Low Intensity** | **Moderate Intensity** | **High Intensity** |
| --- | --- | --- | --- |
| Atorvastatin |  | <40 mg | ≥40 mg |
| Rosuvastatin |  | <20 mg | ≥20 mg |
| Lovastatin | <40 mg | ≥40 mg |  |
| Simvastatin | <20 mg | 20–79 mg before 2014 ≥80 mg in 2014 | ≥80 mg before 2014 |
| Pravastatin | <40 mg | ≥40 mg |  |
| Fluvastatin (XL) |  | ≥80 mg |  |
| Fluvastatin | <80 mg | ≥80 mg |  |
| Pitavastatin | <2 mg | ≥2mg |  |

Data modified from the 2013 American College of Cardiology and American Heart Association joint guidelines, Circulation. 2013;129(25 suppl 2):S1-S45**.**

Supplementary Table 2. Summary of codes for disease categorization

| **Code brief description** | **Code** | **Description** |
| --- | --- | --- |
| ACS; MI; CVD | 410.xx | Acute myocardial infarction of anterolateral wall |
| STEMI; MI; ACS; CVD | 410.0 410.61410.81 | ST elevation of MI |
| NSTEMI; MI; ACS; CVD | 410.71 | Non-ST elevation of MI |
| USTEMI; MI; ACS; CVD | 410.91 | Unspecified ST elevation of MI |
| ACS; ANGINA | 411.1x | Intermediate coronary syndrome |
| DM | 250.xx | Diabetes |
| AF | 427.31 | Atrial fibrillation |
| HYPT | 401.xx | Essential hypertension |
| HYPT; CVD; VHD | 402.xx | Hypertensive heart disease |
| HYPT; CKD/RI | 403.xx | Hypertensive heart disease |
| HYPT; CVD; CKD/RI | 404.xx | Hypertensive heart and chronic kidney disease |
| HYPT | 405.xx | Secondary hypertension |
| DYSPA | 272.xx | Disorders of lipoid metabolism, including hypercholesteremia, hyperglyceridemia, hyperlipidemia, hyperchylomicronemia, lipoprotein deficiencies, lipodystrophy, lipidoses |
| OLD MI | 412 | Old myocardial infarction |
| Unstable angina; ACS; CVD | 411.1 | Intermediate coronary syndrome |
| Unstable angina | 411.81 | Acute coronary occlusion without myocardial infarction |
| STROKE | 431.xx | Intracerebral hemorrhage |
| STROKE | 433.xx | Occlusion and stenosis of precerebral arteries |
| STROKE | 434.xx | Occlusion of cerebral arteries |
| HF; CVD | 428.xx | Heart failure |
| PCI; REVAZ | 0.66 | Percutaneous transluminal coronary angioplasty [PTCA] |
| PCI; REVAZ | 36.06 | Insertion of non-drug-eluting coronary artery stent(s) |
| PCI; REVAZ | 36.07 | Insertion of drug-eluting coronary artery stent(s) |
| PCI; REVAZ | 17.55 | Transluminal coronary atherectomy |
| PCI; REVAZ | 0.40 | Procedures on single vessel/Number of vessels, unspecified |
| PCI; REVAZ | 0.41 | Procedure on two vessels |
| PCI; REVAZ | 0.42 | Procedure on three vessels |
| PCI; REVAZ | 0.43 | Procedure on four or more vessels |
| PCI; REVAZ | 0.44 | Procedure on vessel bifurcation |
| PCI; REVAZ | 0.45 | Insertion of one vascular stent (or number of stents unspecified) |
| PCI; REVAZ | 0.46 | Insertion of two vascular stents |
| PCI; REVAZ | 0.47 | Insertion of three vascular stents |
| PCI; REVAZ | 0.48 | Insertion of four or more vascular stents |
| PCI; REVAZ | 92980 | Transcatheter placement of an intracoronary stent(s), percutaneous, with or without other therapeutic intervention, any method; single vessel |
| PCI; REVAZ | 92981 | Transcatheter placement of an intracoronary stent(s), percutaneous, with or without other therapeutic intervention, any method; each additional vessel |
| PCI; REVAZ | 92982 | Percutaneous transluminal coronary balloon angioplasty; single vessel |
| PCI; REVAZ | 92984 | Percutaneous transluminal coronary balloon angioplasty; each additional vessel |
| PCI; REVAZ | 92995 | Percutaneous transluminal coronary atherectomy, by mechanical or other method, with or without balloon angioplasty; single vessel |
| PCI; REVAZ | 92996 | Percutaneous transluminal coronary atherectomy, by mechanical or other method, with or without balloon angioplasty; each additional vessel |
| PCI; REVAZ | 92920 | Percutaneous transluminal coronary angioplasty; single major coronary artery or branch |
| PCI; REVAZ | 92921 | Percutaneous transluminal coronary angioplasty; each additional branch of a major coronary artery |
| PCI; REVAZ | 92924 | Percutaneous transluminal coronary atherectomy, with coronary angioplasty when performed; single major coronary artery or branch |
| PCI; REVAZ | 92925 | Percutaneous transluminal coronary atherectomy, with coronary angioplasty when performed; each additional branch of a major coronary artery |
| PCI; REVAZ | 92928 | Percutaneous transcatheter placement of intracoronary stent(s), with coronary angioplasty when performed; single major coronary artery or branch |
| PCI; REVAZ | 92929 | Percutaneous transcatheter placement of intracoronary stent(s), with coronary angioplasty when performed; each additional branch of a major coronary artery |
| PCI; REVAZ | 92933 | Percutaneous transluminal coronary atherectomy, with intracoronary stent, with coronary angioplasty when performed; single major coronary artery or branch |
| PCI; REVAZ | 92934 | Percutaneous transluminal coronary atherectomy, with intracoronary stent, with coronary angioplasty when performed; each additional branch of a major coronary artery |
| PCI; REVAZ | 92937 | Percutaneous transluminal revascularization of or through coronary artery bypass graft (internal mammary, free arterial, venous), any combination of intracoronary stent, atherectomy and angioplasty, including distal protection when performed; single vessel |
| PCI; REVAZ | 92938 | Percutaneous transluminal revascularization of or through coronary artery bypass graft (internal mammary, free arterial, venous), any combination of intracoronary stent, atherectomy and angioplasty, including distal protection when performed; each additional branch subtended by the bypass graft |
| PCI; REVAZ | 92941 | Percutaneous transluminal revascularization of acute total/subtotal occlusion during acute myocardial infarction, coronary artery or coronary artery bypass graft, any combination of intracoronary stent, atherectomy and angioplasty, including aspiration thrombectomy when performed, single vessel |
| PCI; REVAZ | 92943 | Percutaneous transluminal revascularization of chronic total occlusion, coronary artery, coronary artery branch, or coronary artery bypass graft, any combination of intracoronary stent, atherectomy and angioplasty; single vessel |
| PCI; REVAZ | 92944 | Percutaneous transluminal revascularization of chronic total occlusion, coronary artery, coronary artery branch, or coronary artery bypass graft, any combination of intracoronary stent, atherectomy and angioplasty; each additional coronary artery, coronary artery branch, or bypass graft |
| PCI; REVAZ | C9602 | Percutaneous transluminal coronary atherectomy, with drug eluting intracoronary stent, with coronary angioplasty when performed; single major coronary artery or branch |
| PCI; REVAZ | C9603 | Percutaneous transluminal coronary atherectomy, with drug-eluting intracoronary stent, with coronary angioplasty when performed; each additional branch of a major coronary artery |
| PCI; REVAZ | C9604 | Percutaneous transluminal revascularization of or through coronary artery bypass graft (internal mammary, free arterial, venous), any combination of drug-eluting intracoronary stent, atherectomy and angioplasty, including distal protection when performed; single vessel |
| PCI; REVAZ | C9605 | Percutaneous transluminal revascularization of or through coronary artery bypass graft (internal mammary, free arterial, venous), any combination of drug-eluting intracoronary stent, atherectomy and angioplasty, including distal protection when performed; each additional branch subtended by the bypass graft |
| PCI; REVAZ | C9606 | Percutaneous transluminal revascularization of acute total/subtotal occlusion during acute myocardial infarction, coronary artery or coronary artery bypass graft, any combination of drug-eluting intracoronary stent, atherectomy and angioplasty, including aspiration thrombectomy when performed, single vessel |
| PCI; REVAZ | C9607 | Percutaneous transluminal revascularization of chronic total occlusion, coronary artery, coronary artery branch, or coronary artery bypass graft, any combination of drug-eluting intracoronary stent, atherectomy and angioplasty; single vessel |
| PCI; REVAZ | C9608 | Percutaneous transluminal revascularization of chronic total occlusion, coronary artery, coronary artery branch, or coronary artery bypass graft, any combination of drug-eluting intracoronary stent, atherectomy and angioplasty; each additional coronary artery, coronary artery branch, or bypass graft |
| PCI; REVAZ | G0290 | Transcatheter placement of a drug eluting intracoronary stent(s), percutaneous, with or without other therapeutic intervention, any method; single vessel |
| PCI; REVAZ | G0291 | Transcatheter placement of a drug eluting intracoronary stent(s), percutaneous, with or without other therapeutic intervention, any method; each additional vessel |
| PCI; REVAZ | C9600 | Percutaneous transcatheter placement of drug eluting intracoronary stent(s), with coronary angioplasty when performed; single major coronary artery or branch |
| PCI; REVAZ | C9601 | Percutaneous transcatheter placement of drug-eluting intracoronary stent(s), with coronary angioplasty when performed; each additional branch of a major coronary artery |
| CABG; REVAZ | 36.10 | Aortocoronary bypass, unspecified |
| CABG; REVAZ | 36.11 | Aortocoronary bypass of 1 coronary artery |
| CABG; REVAZ | 36.12 | Aortocoronary bypass of 2 coronary arteries |
| CABG; REVAZ | 36.13 | Aortocoronary bypass of 3 coronary arteries |
| CABG; REVAZ | 36.14 | Aortocoronary bypass of 4 or more coronary arteries |
| CABG; REVAZ | 36.15 | Single internal mammary-coronary artery bypass |
| CABG; REVAZ | 36.16 | Double internal mammary-coronary artery bypass |
| CABG; REVAZ | 36.17 | Abdominal-coronary artery bypass |
| CABG; REVAZ | 36.19 | Other bypass anastomosis for heart revascularization |
| CABG; REVAZ | 33508 | Endoscopy, surgical, including video-assisted harvest of vein(s) for coronary artery bypass procedure |
| CABG; REVAZ | 33510 | Coronary artery bypass, vein only; single coronary venous graft |
| CABG; REVAZ | 33511 | Coronary artery bypass, vein only; two coronary venous grafts |
| CABG; REVAZ | 33512 | Coronary artery bypass, vein only; three coronary venous grafts |
| CABG; REVAZ | 33513 | Coronary artery bypass, vein only; four coronary venous grafts |
| CABG; REVAZ | 33514 | Coronary artery bypass, vein only; five coronary venous grafts |
| CABG; REVAZ | 33516 | Coronary artery bypass, vein only; six or more coronary venous grafts |
| CABG; REVAZ | 33517 | Coronary artery bypass, using venous graft(s) and arterial graft(s); single vein graft |
| CABG; REVAZ | 33518 | Coronary artery bypass, using venous graft(s) and arterial graft(s); two venous grafts |
| CABG; REVAZ | 33519 | Coronary artery bypass, using venous graft(s) and arterial graft(s); three venous grafts |
| CABG; REVAZ | 33521 | Coronary artery bypass, using venous graft(s) and arterial graft(s); four venous grafts |
| CABG; REVAZ | 33522 | Coronary artery bypass, using venous graft(s) and arterial graft(s); five venous grafts |
| CABG; REVAZ | 33523 | Coronary artery bypass, using venous graft(s) and arterial graft(s); six or more venous grafts |
| CABG; REVAZ | 33533 | Coronary artery bypass, using arterial graft(s); single arterial graft |
| CABG; REVAZ | 33534 | Coronary artery bypass, using arterial graft(s); two coronary arterial grafts |
| CABG; REVAZ | 33535 | Coronary artery bypass, using arterial graft(s); three coronary arterial grafts |
| CABG; REVAZ | 33536 | Coronary artery bypass, using arterial graft(s); four or more coronary arterial grafts |
| CABG; REVAZ | S2205 | Minimally invasive direct coronary artery bypass surgery involving mini-thoracotomy or mini-sternotomy surgery, performed under direct vision; using arterial graft(s), single coronary arterial graft |
| CABG; REVAZ | S2206 | Minimally invasive direct coronary artery bypass surgery involving mini-thoracotomy or mini-sternotomy surgery, performed under direct vision; using arterial graft(s), two coronary arterial grafts |
| CABG; REVAZ | S2207 | Minimally invasive direct coronary artery bypass surgery involving mini-thoracotomy or mini-sternotomy surgery, performed under direct vision; using venous graft only, single coronary venous graft |
| CABG; REVAZ | S2208 | Minimally invasive direct coronary artery bypass surgery involving mini-thoracotomy or mini-sternotomy surgery, performed under direct vision; using single arterial and venous graft(s), single venous graft |
| CABG; REVAZ | S2209 | Minimally invasive direct coronary artery bypass surgery involving mini-thoracotomy or mini-sternotomy surgery, performed under direct vision; using two arterial grafts and single |
| CKD/RI | 274.1 | Gouty nephropathy, unspecified |
| CKD/RI | 274.11 | Uric acid nephrolithiasis |
| CKD/RI | 274.19 | Other gouty nephropathy |
| CKD/RI; HYPT | 403.xx | Hypertensive chronic kidney disease |
| CKD/RI; HYPT; CVD | 404.xx | Hypertensive heart and kidney disease |
| CKD/RI | 582.xx | Chronic glomerulonephritis |
| CKD/RI | 585.xx | Chronic renal failure |
| CKD/RI | 590.00 | Chronic pyelonephritis without lesion of renal medullary necrosis |
| CKD/RI | 590.01 | Chronic pyelonephritis with lesion of renal medullary necrosis |
| CKD/RI | 753.0 | Renal agenesis and dysgenesis |
| CKD/RI | 753.1x | Cystic kidney disease |
| CKD/RI | 753.3 | Other specified anomalies of kidney |
| CVD | 391.x | Rheumatic fever with heart involvement |
| CVD | 392.0 | Rheumatic chorea with heart involvement |
| CVD | 393 | Chronic rheumatic pericarditis |
| CVD; VHD | 394.x | Diseases of mitral valve |
| CVD; VHD | 395.x | Diseases of aortic valve |
| CVD; VHD | 396.x | Diseases of mitral and aortic valves |
| CVD; VHD | 397.x | Diseases of other endocardial structures |
| CVD | 398.xx | Other rheumatic heart disease |
| CVD; HYPT | 402.xx | Hypertensive heart disease |
| CVD; HYPT | 404.00 | Hypertensive heart and chronic kidney disease, malignant, without heart failure and with chronic kidney disease stage I through stage IV, or unspecified |
| CVD; HYPT | 404.01 | Hypertensive heart and chronic kidney disease, malignant, with heart failure and with chronic kidney disease stage I through stage IV, or unspecified |
| CVD; HYPT | 404.03 | Hypertensive heart and chronic kidney disease, malignant, with heart failure and with chronic kidney disease stage V or end stage renal disease |
| CVD; HYPT | 404.10 | Hypertensive heart and chronic kidney disease, benign, without heart failure and with chronic kidney disease stage I through stage IV, or unspecified |
| CVD; HYPT | 404.11 | Hypertensive heart and chronic kidney disease, benign, with heart failure and with chronic kidney disease stage I through stage IV, or unspecified |
| CVD; HYPT | 404.13 | Hypertensive heart and chronic kidney disease, benign, with heart failure and chronic kidney disease stage V or end stage renal disease |
| CVD; HYPT | 404.90 | Hypertensive heart and chronic kidney disease, unspecified, without heart failure and with chronic kidney disease stage I through stage IV, or unspecified |
| CVD; HYPT | 404.91 | Hypertensive heart and chronic kidney disease, unspecified, with heart failure and with chronic kidney disease stage I through stage IV, or unspecified |
| CVD; HYPT | 404.93 | Hypertensive heart and chronic kidney disease, unspecified, with heart failure and chronic kidney disease stage V or end stage renal disease |
| CVD | 413.x | Angina pectoris |
| CVD; VHD | 414.xx | Other forms of chronic ischemic heart disease |
| CVD | 415.0 | Acute cor pulmonale |
| CVD | 416.x | Chronic pulmonary heart disease |
| CVD; VHD | 420.xx | Acute pericarditis |
| CVD | 421.x | Acute and subacute endocarditis |
| CVD | 422.xx | Acute myocarditis |
| CVD | 423.x | Other diseases of pericardium |
| CVD; VHD | 424.xx | Other diseases of endocardium |
| CVD | 425.xx | Cardiomyopathy |
| CVD | 426.xx | Conduction disorders |
| CVD; AF | 427.xx | Cardiac dysrhythmias |
| CVD | 429.xx | Ill-defined descriptions and complications of heart disease |
| VHD | 391.1 | Acute rheumatic endocarditis |
| REVAZ | 36.03 | Open chest coronary artery angioplasty |
| REVAZ | 36.04 | Intracoronary artery thrombolytic infusion |
| REVAZ | 36.09 | Other removal of coronary artery obstruction |
| REVAZ | 36.2 | Heart revascularization by arterial implant |
| REVAZ | 36.3x | Other heart revascularization |
| REVAZ | 33999 | Unlisted procedure, cardiac surgery |
| REVAZ | 33140 | Transmyocardial laser revascularization, by thoracotomy; (separate procedure) |
| REVAZ | 33141 | Transmyocardial laser revascularization, by thoracotomy; performed at the time of other open cardiac procedure(s) (List separately in addition to code for primary procedure) |

Supplementary Table 3. Cardiovascular outcomes during the follow-up period

| **Cardiovascular Outcomes^a^** | **Number of Cases** | **Total number of Days for Time to Event or Censoring** | **Incidence Rate^b^ (N with ≥1 event/days from discharge to event, per 1000 person-years)** |
| --- | --- | --- | --- |
| Recurrent ACS admission | 740 | 6422 | **115.2** |
| MI | 476 | 6561 | **72.5** |
| Unstable angina | 408 | 6613 | **61.7** |
| Stroke admission | 173 | 6762 | **25.6** |
| Heart failure admission^c^ | 715 | 6497 | **110.0** |
| Revascularization admission | 499 | 6529 | **76.4** |
| Percutaneous coronary intervention | 365 | 6610 | **55.2** |
| Coronary artery bypass graft | 123 | 6756 | **18.2** |
| Valvular heart disease^c^ | 337 | 6668 | **50.5** |
| Cardiovascular-specific deaths^d^ | 78 | 6827 | **11.4** |

Abbreviations: ACS, acute coronary syndrome; MI, myocardial infarction.

^a^ Reported in person-years and defined as the number of patients with a cardiovascular-related inpatient admission or death during the variable follow-up period divided by the total number of days between the index date and the cardiovascular-related inpatient admission or death date, or censoring date for patients without an event (earliest date for death, end of continuous enrollment, 12-months post-index, or end of study). For patients with multiple cardiovascular-related inpatient admissions during the follow-up period, the first inpatient date and/or emergency room date was selected. In addition, cardiovascular outcomes were measured as time-to-event variables which were summarized by the incidence rate of the endpoint.

^b^ Incidence rates were calculated by dividing the number of patients experiencing at least 1 occurrence of an event during follow-up by the total number of days between the ACS discharge date and the event of interest, or censoring at the end of follow-up for patients without an event. For patients with multiple cardiovascular-specific inpatient admissions during the follow-up period, the first inpatient date and/or emergency room date was selected. **Rates were multiplied by 1000 to present in 1000 person-years.**

^c^ Patients with baseline heart failure or valvular heart disease were evaluated only for the outcome of heart failure or valvular heart disease inpatient admission, respectively.

^d^ Defined as inpatient record carrying a cardiovascular primary diagnosis code and an inpatient death discharge.

Supplementary Table 4. Mean PPPM all-cause and cardiovascular-specific healthcare cost outcomes during the follow-up period

| **US$** | **All-Cause Costs** | **Cardiovascular-Specific Costs^a^** |
| --- | --- | --- |
| Any inpatient admissions | 1161 | 614 |
| Outpatient services |  |  |
| Emergency room visits | 59 | 26 |
| Physician office visits | 83 | 32 |
| Laboratory | 61 | 22 |
| Radiology | 97 | 28 |
| Other | 994 | 388 |
| Outpatient pharmacy | 414 | 280 |
| Total medical costs | 2456 | 1111 |
| Total healthcare costs | 2870 | 1391 |

Abbreviation: ICD-9-CM, International Classification of Diseases, Ninth Revision, Clinical Modification; PPPM, per patient per month.

Costs presented as PPPM due to the variable follow-up period.

^a^ Cardiovascular-specific healthcare utilization and costs were defined by pulling medical claims with ICD-9-CM diagnosis codes for a cardiovascular condition and pharmacy claims for cardiovascular medications. For inpatient admissions, the diagnosis code must be in the primary diagnosis position on the claim.

Supplementary Fig. 1. Study period


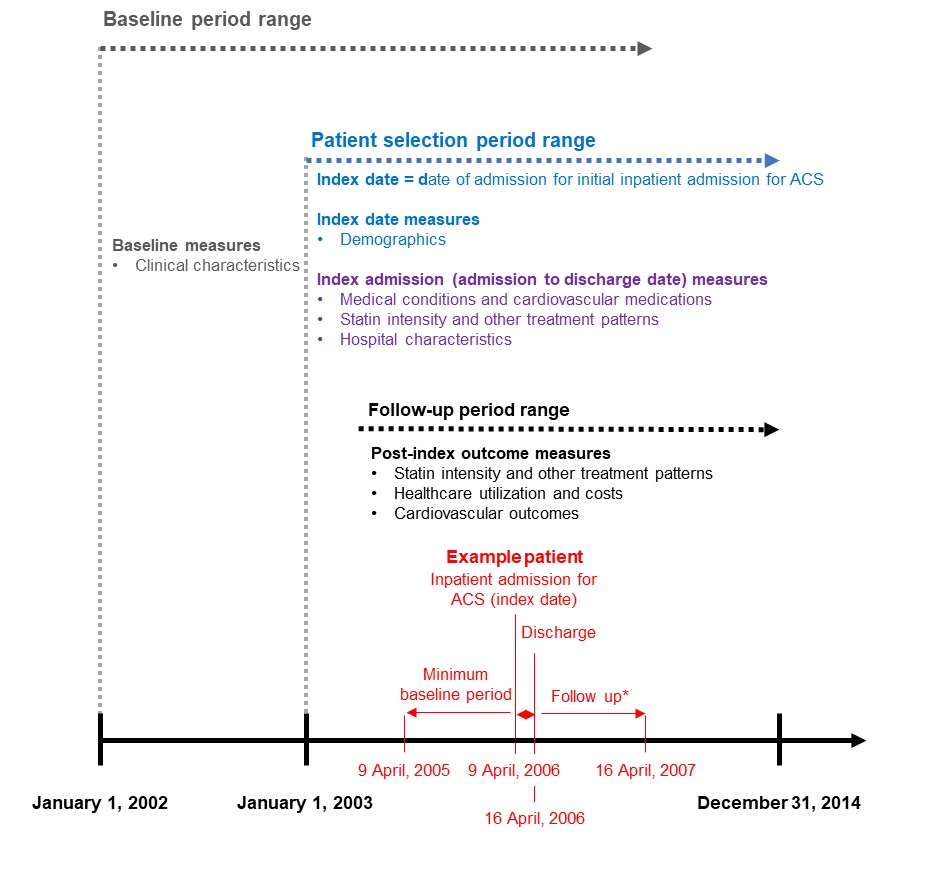


Abbreviation: ACS, acute coronary syndrome.
***The follow-up period extends from the discharge date until the earliest of a) 12 months post discharge; b) inpatient death; c) dis-enrollment from health insurance, or d) end of study period.**

Supplementary Fig. 2. Index hospitalization diagnosis and cardiovascular treatment patterns in all patients (N = 7802)


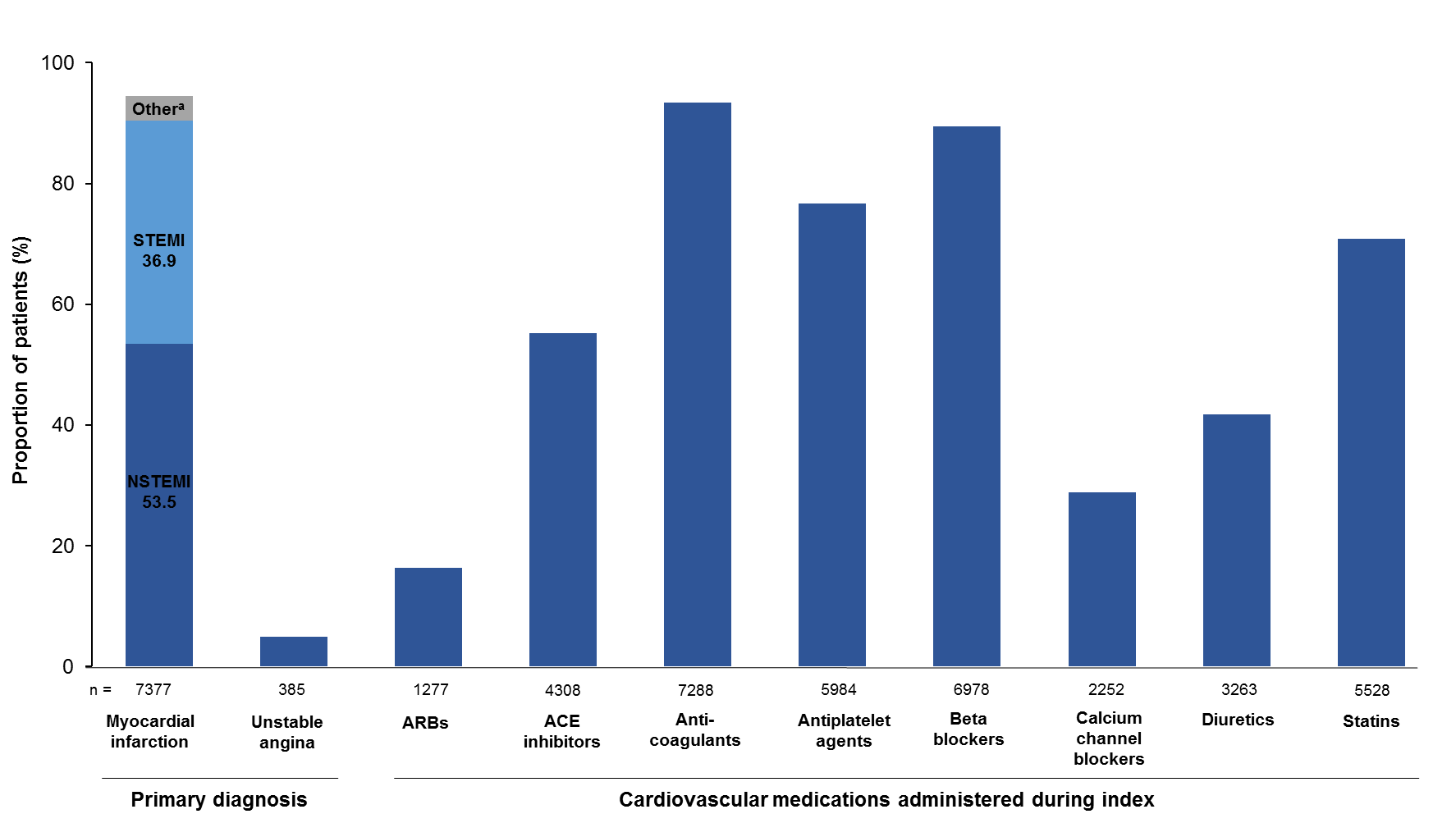


Abbreviations: ACE, angiotensin converting enzyme; ARBs, angiotensin receptor blockers; MI, myocardial infarction; NSTEMI, non-ST-segment elevation myocardial infarction; STEMI, ST-segment elevation myocardial infarction.

Total number of patients included in the analysis = 7802.

^a^ Includes other MI, n = 313; 4.0%.

Supplementary Fig. 3. Statin treatment patterns during the follow-up period by the overall cohort and by pre- and post-2006 ACS events


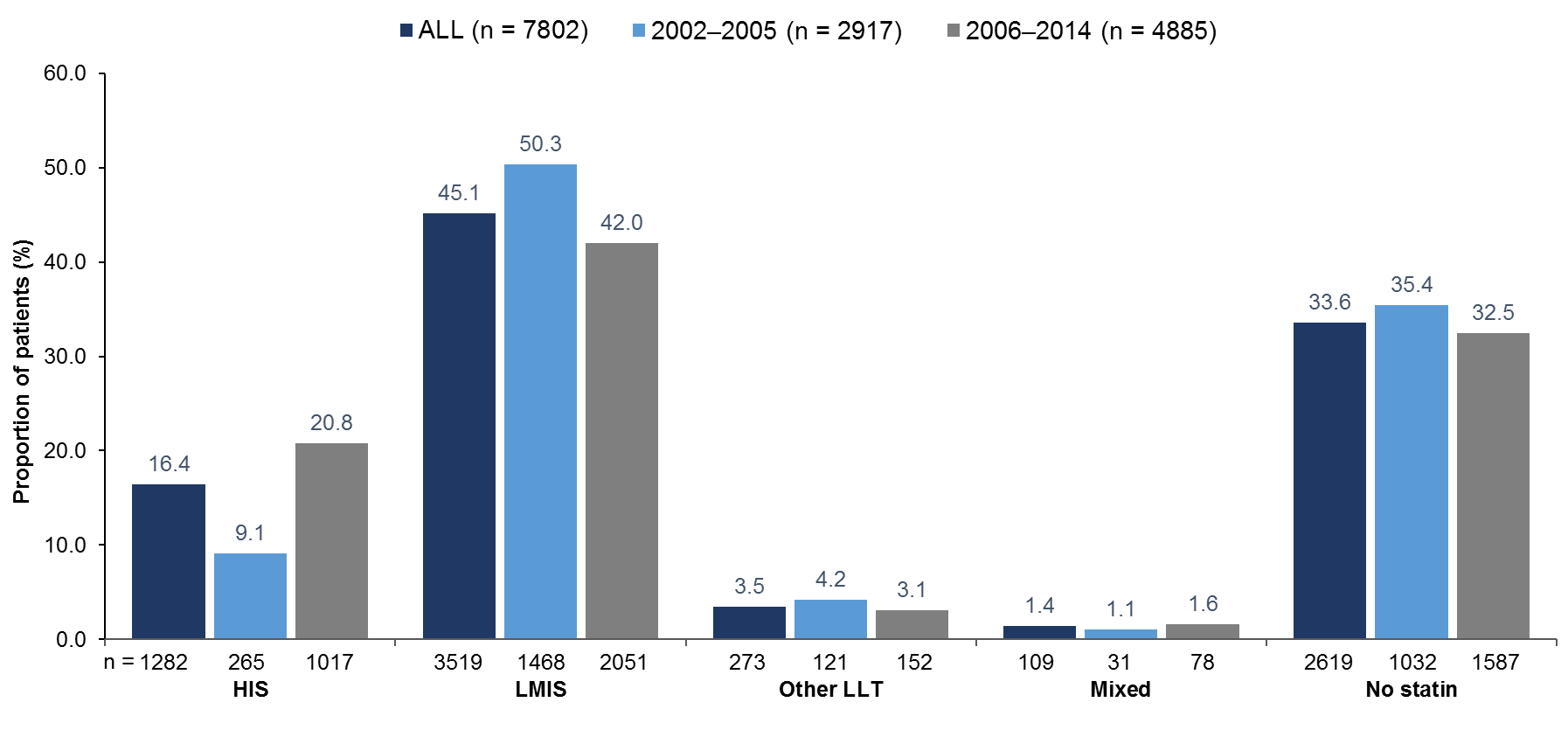


Abbreviations: ACS, acute coronary syndrome; HIS, high intensity statin; LLT, lipid-lowering therapy; LMIS, low- to-moderate-intensity statin.
